# Laser-scribed graphene nanofiber decorated with oil palm lignin capped silver nanoparticles: a green biosensor

**DOI:** 10.1038/s41598-021-85039-2

**Published:** 2021-03-09

**Authors:** Melvin Jia Yong Tai, Veeradasan Perumal, Subash C. B. Gopinath, Pandian Bothi Raja, Mohamad Nasir Mohamad Ibrahim, Iffah Najihah Jantan, Nur Syahirah Husna Suhaimi, Wei-Wen Liu

**Affiliations:** 1 Centre of Innovative Nanostructures & Nanodevices (COINN), Universiti Teknologi PETRONAS (UTP), Seri Iskandar, Perak Malaysia; 2 Mechanical Engineering Department, Universiti Teknologi PETRONAS (UTP), Seri Iskandar, Perak Malaysia; 3 Institute of Nano Electronic Engineering, Universiti Malaysia Perlis (UniMAP), Kangar, Perlis Malaysia; 4 Faculty of Chemical Engineering Technology, Universiti Malaysia Perlis (UniMAP), Arau, Perlis Malaysia; 5 School of Chemical Sciences, Universiti Sains Malaysia, Gelugor, Penang, Malaysia

**Keywords:** Biochemistry, Biomarkers, Nanoscience and technology

## Abstract

Tuberculosis (TB), caused by *Mycobacterium tuberculosis* (*M. tuberculosis*), requires a high level of attention and is one of the most infectious diseases in the air. Present methods of diagnosing TB remain ineffective owing to their low sensitivity and time consumption. In this study, we produced a green graphene nanofiber laser biosensor (LSG-NF) decorated with oil palm lignin-based synthetic silver nanoparticles (AgNPs). The resulting composite morphology was observed by field-emission scanning electron microscopy and transmission electron microscopy, which revealed the effective adaptation of the AgNPs to the LSG-NF surface. The successful attachment of AgNPs and LSG-NFs was also evident from X-ray diffraction and Raman spectroscopy studies. In order to verify the sensing efficiency, a selective DNA sample captured on AgNPs was investigated for specific binding with M.tb target DNA through selective hybridisation and mismatch analysis. Electrochemical impedance studies further confirmed sensitive detection of up to 1 fM, where a detection limit of 10^−15^ M was obtained by estimating the signal-to-noise ratio (*S/N* = 3:1) as 3σ. Successful DNA immobilisation and hybridisation was confirmed by the detection of phosphorus and nitrogen peaks based on X-ray photoelectron spectroscopy and Fourier-transform infrared spectroscopy. The stability and repeatability of the analysis were high. This approach provides an affordable potential sensing system for the determination of *M. tuberculosis* biomarker and thus provides a new direction in medical diagnosis.

## Introduction

Tuberculosis (TB) is considered on par with HIV/AIDS as a major disease^1^, affecting billions of people and causing millions of deaths each year. *Mycobacterium tuberculosis* (*M. tuberculosis*) is the key pathogen responsible for TB. The World Health Organization (WHO) adopted the ‘End TB Strategy’ to eliminate this disease by 2035 and obtained a positive response from most countries^2^. These countries have introduced a variety of new approaches to reduce TB epidemics. However, the current techniques for diagnosing this disease suffer from low sensitivity and require prolonged times to obtain correct results^3^. Rapid, cost-effective, and high-sensitivity biosensors are therefore required for the effective detection of TB in clinical samples^4^.

Carbon-based nanomaterials such as graphene, fullerene, and carbon nanotubes (CNTs) have played an important role in various applications owing to their specific properties^5^. The design of these carbon nanomaterials has a direct impact on their electron transfer rate and therefore affects their analytical efficiency^6^ in areas such as electrochemical sensing^7^. Interestingly, graphene has received much attention in electrochemical research. These carbon-based materials possess many beneficial properties, such as high thermal and electrical conductivity. Previous reports indicate that graphene displays excellent electrochemical properties when it adopts a porous 3D network pattern, enabling the mass transfer of charged electrons. While graphene provides outstanding functionality, graphene manufacturing processes are costly and complicated^5^. Therefore, the industrial production of graphene devices in a cost-effective manner is mandatory.

Previous research has reported a simpler and efficient method of graphene synthesis by penetrating a commercial polyimide (PI) film with a CO_2_ laser in an ambient graphene-scribing laser (LSG) setting^8^. Surprisingly, the recorded process generated a porous 3D network pattern, affording highly defective graphene, but offering high electrical conductivity and stability, resulting in excellent electrochemical performance for sensing applications. A novel graphene product called laser-induced graphene fibre (LIGF) was also obtained by monitoring the amount of radiation energy entering the polyimide film, where the quality of graphitization was evaluated by using a LIGF-formed microsupercapacitor^9^. In addition, several studies have revealed the efficacy of LSG in transporting electrons in biochemical reactions, thus serving as a perfect interface platform for biomolecules^5–7,10^.

The use of graphene as a standalone electrode for sensing devices has been documented, but the electrochemical efficiency of the developed electrodes requires further enhancement^11–13^. The introduction of nanoparticles into such bioelectrodes has therefore become a preferred technique for enhancing the selectivity and sensitivity^14^. As far as economy and conductivity are concerned, copper nanoparticles (CuNPs) are the preferred choice for sensor applications, while custom synthesis conditions and easy oxidation in the absence of capping agents make CuNPs an undesirable material^15^. The ultrafast/facile biosynthesis of gold nanoparticles (AuNPs) and silver nanoparticles (AgNPs) using bio-materials has propelled their applications in sundry research fields within a short period. Due to the lower cost of synthesising AgNPs relative to that of AuNPs and the same excellent results achieved with both species in the biosensor research field, AgNPs were selected as the top material choice for the present study^16–18^.

Chemical reduction of silver metal into AgNPs is a traditional approach in which various synthetic reducing agents including NaBH_4_, sodium citrate, hydrazine, etc., have been successfully employed^19,20^. Although these chemicals produce stable AgNPs with the desired particle size, their toxicity has a huge impact on the environment, thus forcing scientists to find green/compatible replacements. Oil palm-derived lignin has many phenolic hydroxyl groups on its molecular skeleton; thus, lignin has been established as an ideal, green, and potential reducing agent for AgNP synthesis. Our group recently developed an eco-friendly and cost-effective method for synthesising silver nanoparticles (AgNPs) from palm oil lignin. Owing to its biocompatibility, high conductivity, and low toxicity, an increase in the analytical performance of the LSG bioelectrode was predicted^16^.

The current work presents a method of manufacturing graphene nanofibers by laser-scribing after modification with oil palm lignin-capped AgNPs to allow direct bonding with a single strand of DNA for the production of a TB bioelectrode. The bioelectrode is characterised morphologically and structurally, and its selectivity and sensitivity are investigated. Decorated laser graphene nanofiber (LSG-NF-AgNP) bioelectrodes provide an avenue for the generation of versatile and high-sensitivity sensing platforms.

## Results and discussion

**Figure 1:**
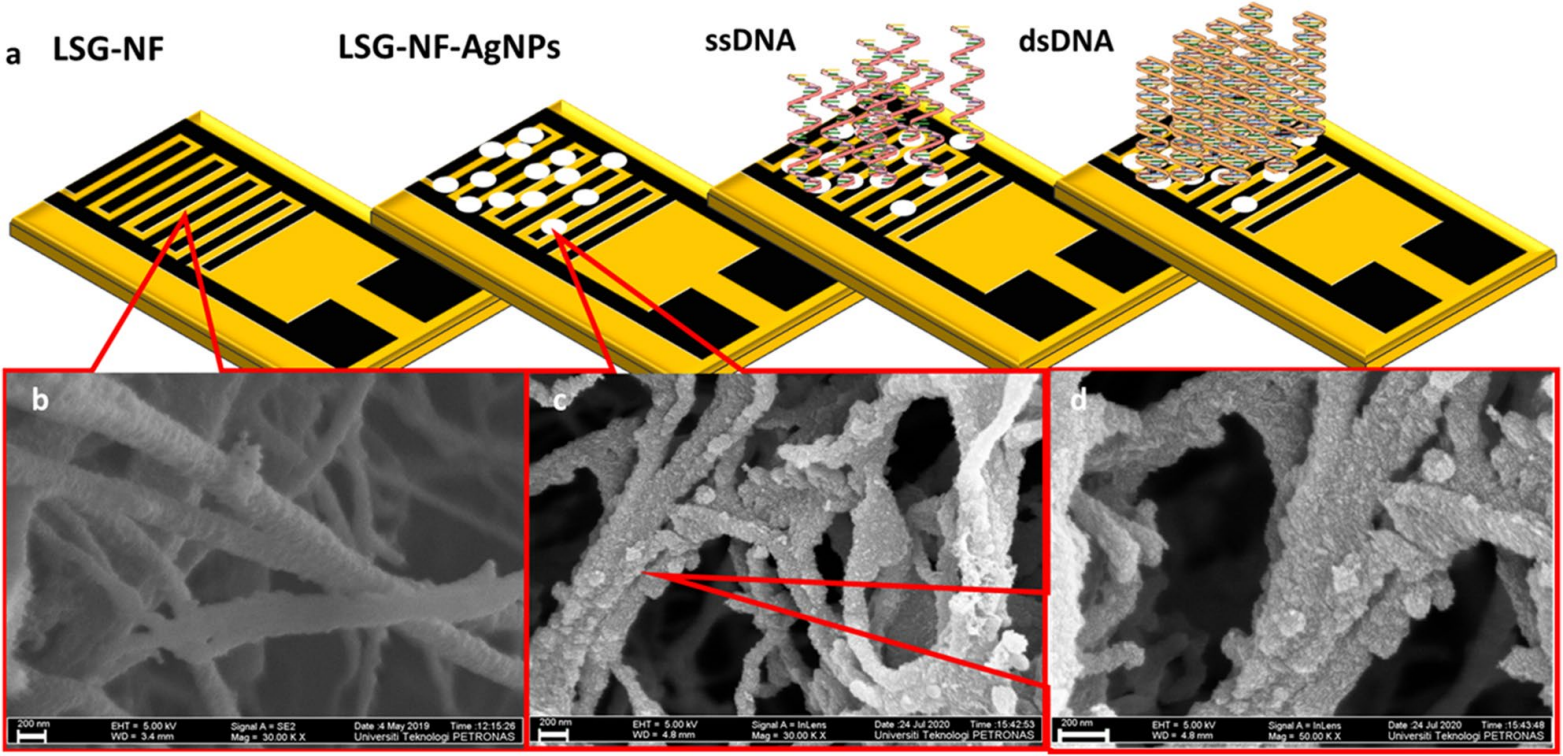
(**a**) Schematic diagram illustrating the process involved in the fabrication of LSG-NF-AgNPs DNA bioelectrode. (**b**) Low-magnification FESEM image showing smooth hair-like structure of synthesized LSG-NF: low- (**c**) and (**d**)- high magnification images of AgNP-decorated LSG-NF. Images show coral-like structure decorated with shiny particles.

The bioelectrode was fabricated by penetrating a PI film with a CO_2_ laser to produce a laser-scribed graphene nanofiber (LSG-NF), where PI works as the substrate on the device. AgNPs were green-synthesised from oil palm lignin (the formation and characterisation are presented in the “Supplementary Information”), which was then added to the LSG-NF by the drop-cast method. The modification creates defects on the LSG-NF and facilitates the accommodation of DNA, increasing the sensitivity of the bioelectrode (Fig. 1a). The surface was characterised by morphological, structural, and chemical analyses to confirm the performance of the biosensor.

### Field-emission scanning electron microscopy (FESEM)

The overall morphology of the LSG-NFs decorated with silver nanoparticles (AgNPs) was characterised by FESEM. Figure 1b,c display the morphological images of LSG-NF obtained before and after introducing the AgNPs using the drop-cast method. Figure 1b shows the structure of the fabricated LSG-NF, which forms a smooth hair-like structure with diameters ranging from 155 to 290 nm. Figure 1c,d show the morphology of the LSG-NF decorated with shiny particles, whereby AgNPs attached on the entire surface grew at a high density to generate a coral-like structure. Clear agglomeration of the AgNPs was observed, indicating homogeneity. Careful observation of the magnified image (Fig. 1d) revealed that the entire surface of the LSG-NF became coarse and the average diameter increased to 300 nm upon accommodation of the AgNPs. This roughened surface affords a higher surface area, providing vacancy defects, which can facilitate the chemisorption of biomolecules on the substrate and permit more facile stacking of DNA during the immobilisation and hybridisation processes^21,22^.

### Transmission electron microscopy (TEM)

**Figure 2:**
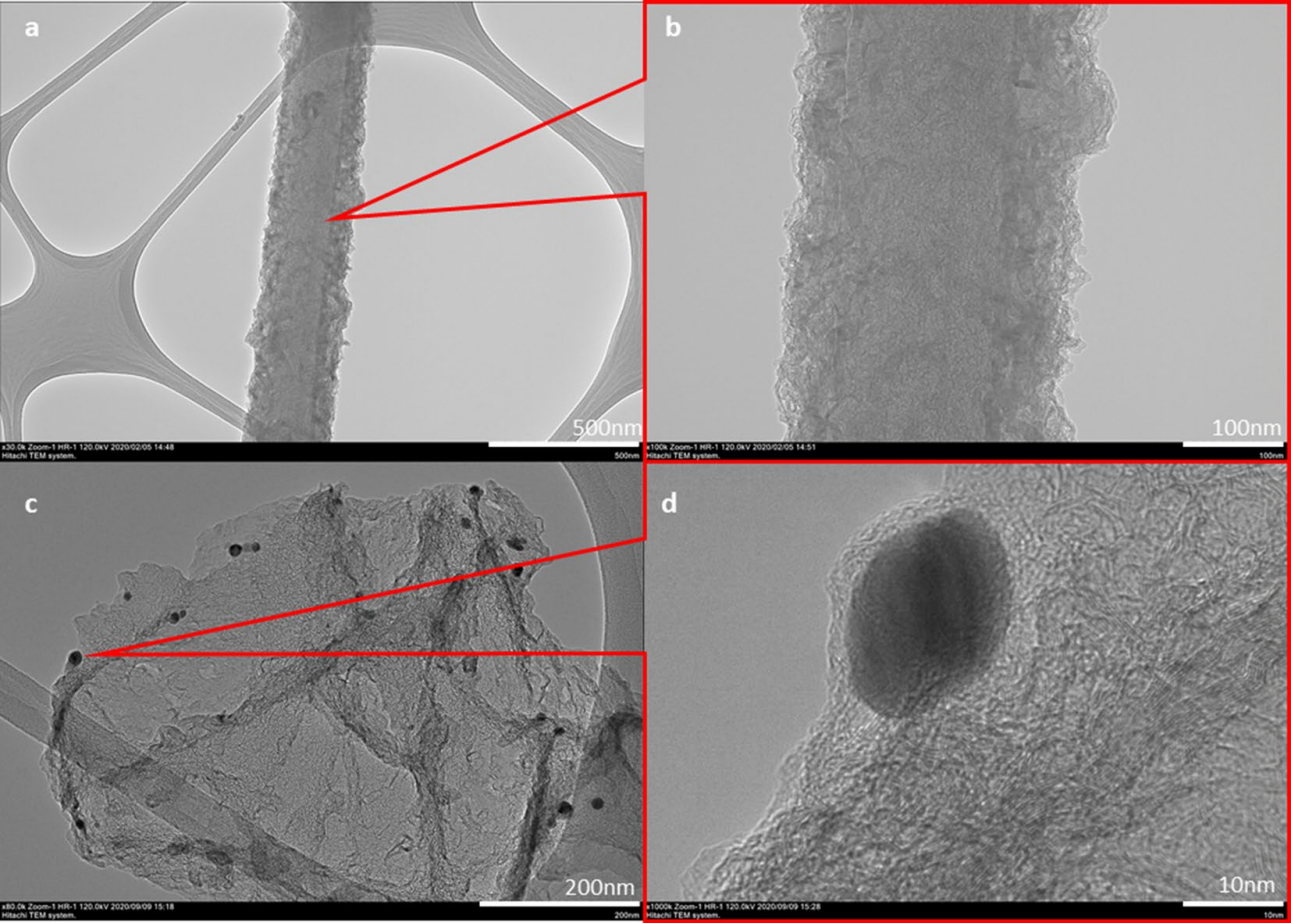
Typical TEM images of (**a**) LSG-NF and (**b**) LSG-NF-AgNPs, (**c**, **d**) shows high magnification TEM images of LSG-NF and LSG-NF-AgNPs, respectively.

The surface morphology of the LSG-NFs and AgNPs was further characterised by TEM. Figure 2 shows the high- and low-magnification TEM images of the LSG-NF fabricated before and after agglomeration of the AgNPs. The low-magnification TEM images of LSG-NF in Fig. 2a reveal the fibre structure of LSG without a hollow core, indicating that the fabricated specimen is not a carbon nanotube. The observation at higher magnification (Fig. 2b) reveals that the diameter of LSG is ~ 290 nm, which is similar to the FESEM results. After introduction of the AgNPs onto LSG, a few black spots were found to be deposited on the graphene layer (Fig. 2c). The higher magnification TEM image in Fig. 2d shows spherical AgNPs as black spots with an average diameter of 19 nm, localized on the edge of LSG. Thus, AgNPs were observed to be randomly attached to the LSG-NF and increased the overall diameter of the specimen^21^.

### X-ray diffraction (XRD)

**Figure 3:**
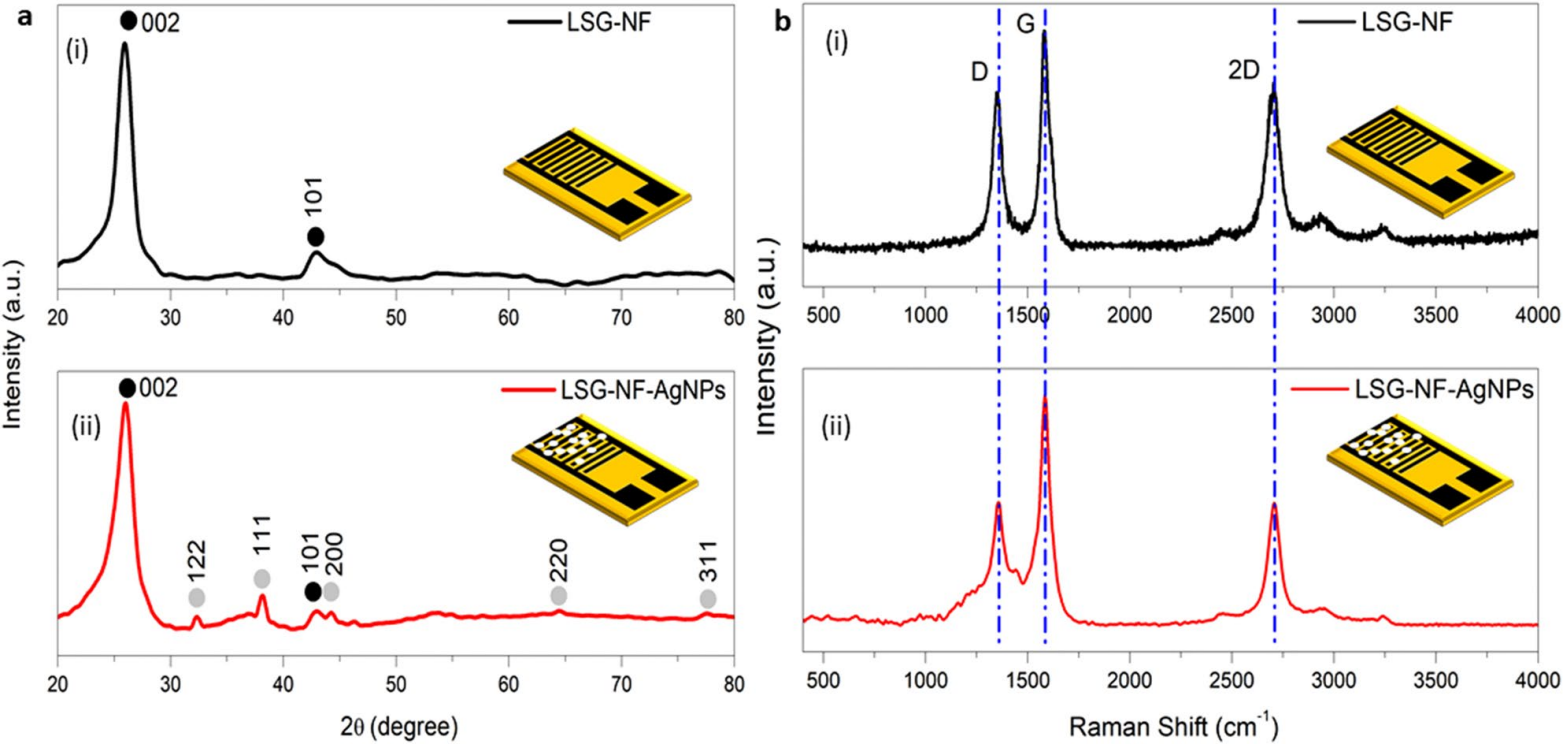
(**a**) X-ray diffraction and (**b**) Raman spectra of LSG-NF, (i) before and (ii) after adding AgNPs.

X-ray diffraction was used to inspect the crystal structure, plane scale, and orientation of the fabricated AgNP-decorated LSG-NFs. The XRD spectra of the AgNPs decorated with LSG-NF are compared with those of plain LSG-NF in Fig. 3a. The XRD patterns presented peaks at 25.96° (002) and 46.26° (101), attributed to graphene (black spectrum). Remarkably, three extra diffraction peaks appeared in the profile of the LSG-NF-AgNPs (red spectrum) relative to that of pure LSG-NF. The diffraction peaks at 33.03°, 38.75°, 44.27°, 64.57°, and 77.44° were assigned to the diffraction lines of the (122), (111), (200), (220), and (311) planes, respectively, which suggests that the AgNPs have a face-centred cubic structure with a crystalline morphology^23–26^. No diffraction peak due to lignin was observed in the XRD pattern, as lignin is amorphous in nature and lacks an ordered structure^27,28^. The sharp and narrow diffraction peaks indicate that LSG-NF is highly crystalline.

### Raman spectroscopy

The Raman spectra of pure LSG-NF and AgNP-decorated LSG-NF are displayed in Fig. 3b. The Raman spectra show typical sharp D, G, and 2D bands at 1358 cm^−1^, 1584 cm^−1^, and 2708 cm^−1^, respectively, indicative of the hexagonal graphene lattice. The D band is known as a disorder or defect band. In both spectra, the D band was relatively intense, indicating imperfect arrangement of the LSG-NF and AgNPs, which allows accommodation of the single-stranded DNA on these substrates and enhances the sensitivity of the biosensor. The intensity ratio of the D and G peaks of LSG-NF (0.75) was slightly reduced to 0.52 upon addition of the AgNPs, indicating that the presence of the AgNPs successfully decreased the defects in LSG-NF^29–31^. The *I*_*2D*_*/I*_*G*_ ratio of LSG-NF was calculated to be 0.79, indicating multilayered graphene. For the LSG-NF-AgNPs, the intensity of all three bands was compared to that of the pure LSG-NF, which showed scattering at a lower Raman shift, possibly due to the chemical bonds formed between the lignin molecules and silver, amino nitrogens, and carboxylate groups.

### X-ray photoelectron spectroscopy (XPS)

**Figure 4:**
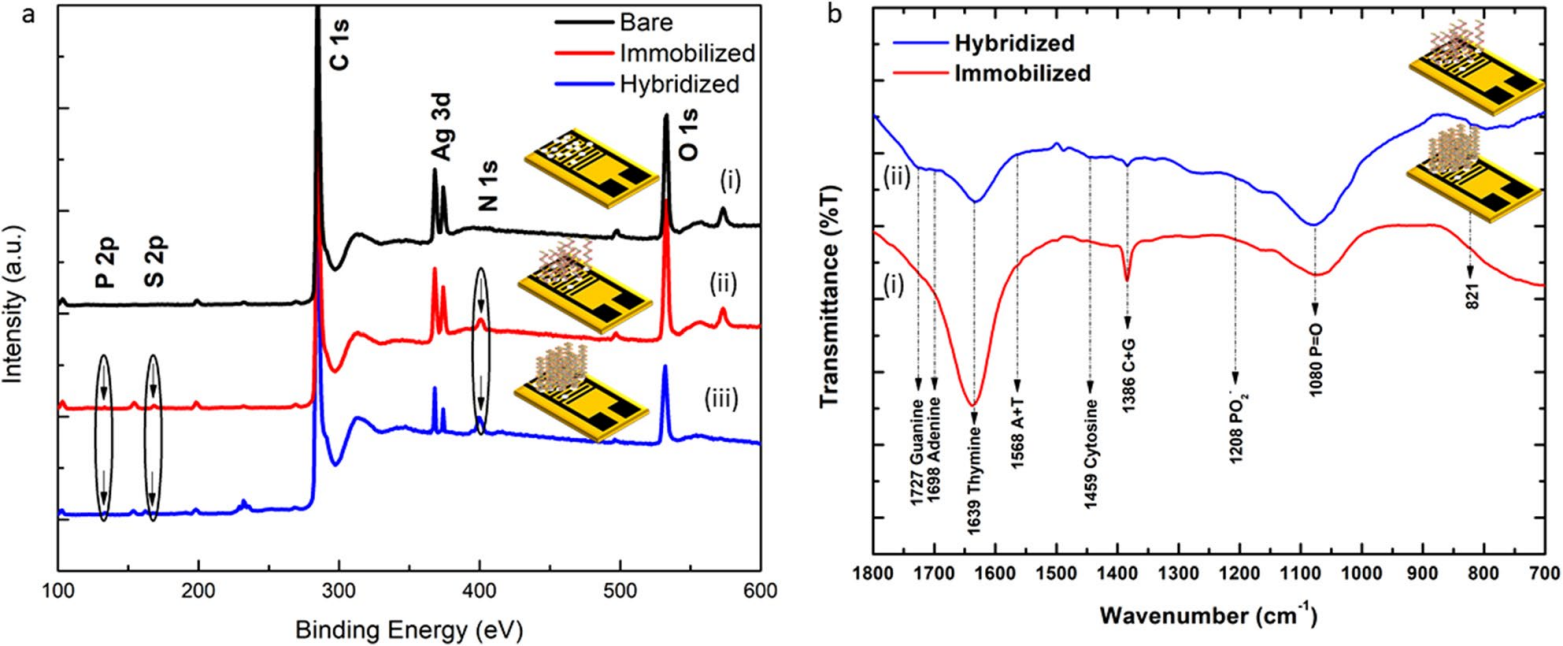
(**a**) Survey scan of XPS core level spectra for (i) LSG-NF-AgNPs, (ii) immobilized (LSG-NF-AgNPs/ssDNA) and hybridized (LSG-NF-AgNPs/dsDNA), (**b**) FTIR spectra of LSG-NF-AgNPs bioelectrode upon (i) immobilization and (ii) hybridization of DNA sequences from *Mycobacterium tuberculosis*. Transmittance region: 700–1800 cm^−1^.

XPS was used to study the elemental composition and chemical state of the elements present on the outermost layer of the AgNP-decorated LSG-NFs and the degree of transformation on the surface upon functionalization for the detection of *M. tuberculosis*. In the full XPS survey scans (Fig. 4a) of all three samples analysed, photoelectron peaks of carbon, oxygen, silver, sulphur, phosphorus, and nitrogen were observed. The binding energy peaks at 284 eV and 532 eV correspond to C1s and O1s, respectively. Ag3d peaks were also observed at 368 and 374 eV. Upon functionalization, the intensity of the C1s and O1s peaks increased slightly, whereas that of the Ag3d state decreased. This phenomenon indicates the effective immobilisation and hybridisation of the developed bio-electrode. S2p peaks were observed at 163 and 168 eV, attributed to the thiol-terminated probe DNA. In addition, two elements (P2p and N1s) were observed after immobilisation and hybridisation. This supports the FTIR results, where phosphorus and nitrogen functional groups are bound on the bio-electrode. It was also found that the binding energy of both the immobilisation and hybridisation peaks changed slightly due to the formation of a bond between the probe DNA and target DNA on the bioelectrode surface. Therefore, the capability of immobilizing and hybridising the bioelectrode was confirmed from the XPS results.

### Confirmation of immobilization and hybridization through Fourier-transform infrared (FTIR) spectroscopy

FTIR analysis provides details on the chemical compounds present on the outermost layer of the AgNPs decorated with LSG-NF after immobilisation and hybridisation of *M. tuberculosis* DNA sequences based on their vibrational signature. The FTIR spectra of the bare LSG-NF, immobilised LSG-NF (probe)-decorated AgNPs, and hybridised LSG-NF (duplex)-decorated AgNPs are shown in Fig. 4b. The chemical bonds in the functional groups of molecules attenuate the transmission of infrared radiation through stretching and bending vibrations. Prior research has generally confirmed that the 700–1800 cm^−1^ range of absorption or transmittance is the fingerprint area for nucleic acids^32,33^. Figure 4b presents a comparison of the FTIR spectra of the immobilised (ssDNA) and hybridised (dsDNA) surfaces, displaying approximately identical transmittance peaks with minor variations. The presence of deoxyribose phosphate in both the probe and duplex spectra was indicated by the peak at 821 cm^−1^ and an insignificant vibrational band. The peak moved from 900 to 1250 cm^−1^ upon hybridization. Additional peaks were observed in the 900–1250 cm^−1^ range, suggesting enhanced symmetric and asymmetric vibrations of the PO_4_^3−^ group of the probe and target DNA derived from the DNA phosphodiester deoxyribose backbone. The FTIR spectra of both ssDNA and the duplex display absorption peaks at 1080 and 1208 cm^−1^, indicating the presence of DNA phosphate classes^34^. The efficacy of immobilisation and hybridisation of the AgNPs on the decorated LSG-NF bioelectrode was confirmed by the peaks at 1460, 1639, 1698, and 1727 cm^−1^, corresponding to the respective functional groups, that is cytosine, thymine, adenine, and guanine^35^. In brief, the effectiveness of functionalization on the bioelectrode was corroborated by the FTIR data.

### Bio-sensing analyses on AgNPs-decorated laser-scribed graphene nanofiber

**Figure 5:**
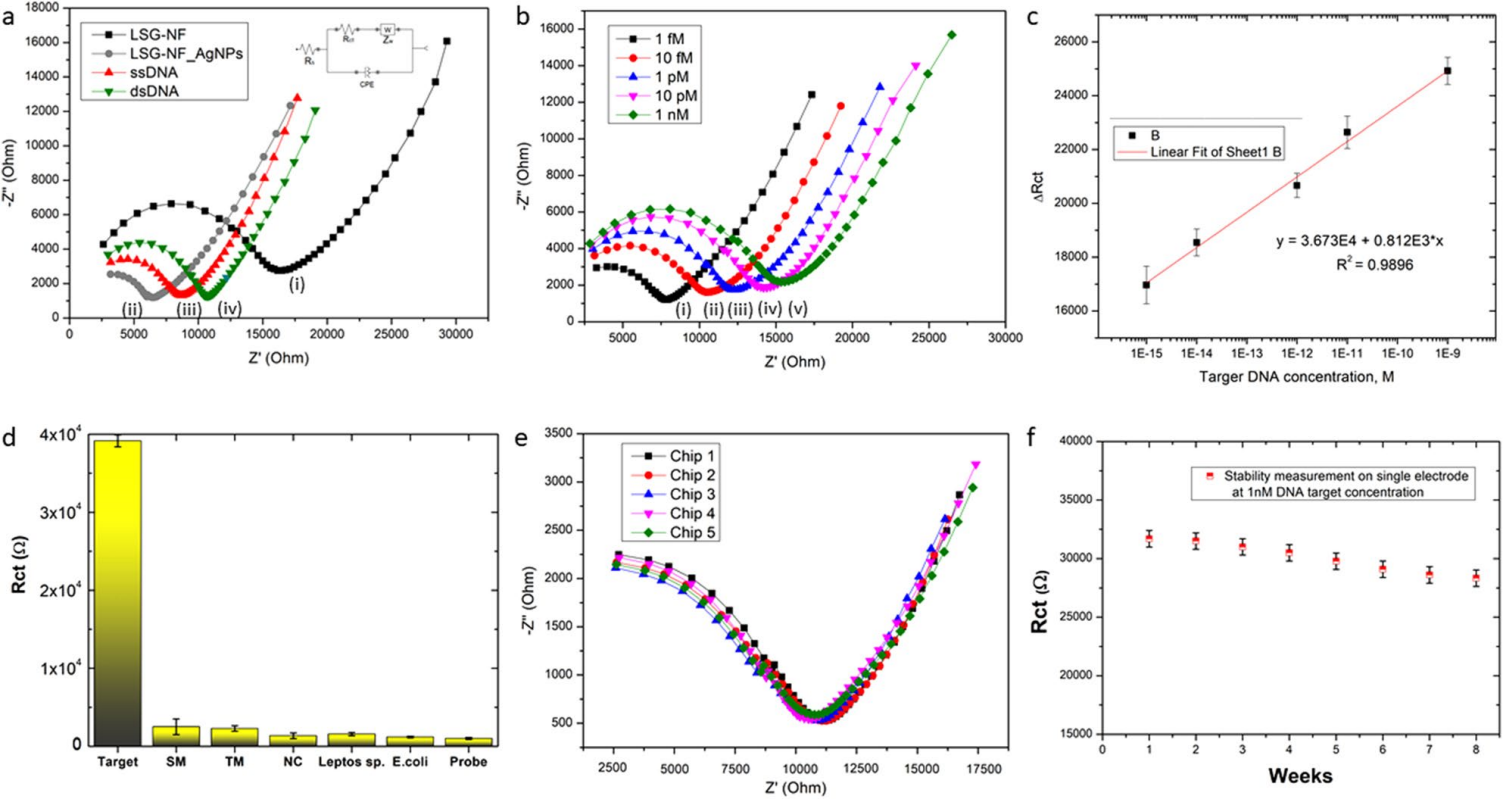
(**a**) Impedimetric curve of (i) LSG-NF, (ii) LSG-NF-AgNP, (ii) immobilized LSG-NF-AgNPs (probe), and (iii) hybridized LSG-NF-AgNPs (duplex) bioelectrode; the inset shows the Randles equivalent circuit, where the parameters *R*_*s*_, *R*_*ct*_, *Z*_*w*_, and CPE represent the bulk solution resistance, charge transfer resistance, Warburg impedance, and constant phase element respectively. (**b**) Impedance spectra of LSG-NF-AgNPs hybridized with different concentrations of complementary target DNA (i–v) 1 fM to 1 nM, (**c**) illustrates the linear regression curve at different concentrations of target DNA with the linear equation: *ΔR*_*ct*_ = 0.812 × 10^3^x + 3.673 × 10^4^, (*R*^*2*^ = 0.9896), (**d**) bar chart showing specificity of the LSG-NF-AgNP bioelectrode against mismatching and cross hybrids, (**e**) reproducibility curve of 5 parallel bioelectrodes fabricated under similar processing conditions, (**f**) stability of LSG-NF-AgNP biosensors.

Electrochemical impedance spectroscopy (EIS) was used to examine the biosensing activity of the AgNPs decorated with LSG-NF. The data acquired over the frequency range of 100 Hz–1 MHz with a 1 × PBS solution (pH 7.4) were used to construct Nyquist plots. Nyquist plots generally show a semicircle at higher frequencies, which denotes the interfacial load transfer resistance (*R*_*ct*_), which correlates to carrier transfer from the bioelectrode to the PBS solution, followed by a linear component at lower frequencies that contributes to the diffusion-limited phase, as shown in Fig. 5a. The acquired Nyquist plot can be expressed by the Randles equivalent circuit (inset), where *R*_*s*_ and *R*_*ct*_ represent the bulk solution resistance and charge transfer resistance, respectively. *Z*_*w*_ represents the Warburg impedance, and CPE is the constant phase element. As seen in Fig. 5a, bare LSG-NF exhibited a higher *R*_*ct*_ (~ 17 K) owing to the presence of imperfections in the LSG-NF. After decoration with the AgNPs, the *R*_*ct*_ was significantly reduced (~ 6 K), indicating that the bare system undergoes excellent electron transfer to the PBS solution owing to its wide surface area^29^. After the immobilisation phase, the *R*_*ct*_ increased to ~ 8.5 K, leading to the adsorption and immobilisation of the ssDNA by the Ag-SH bond on the surface, slowing the diffusion of the electrolyte to the system surface. The improvement in *R*_*ct*_ indicates effective immobilisation of the DNA probe and reduced electrostatic repulsion, attributed to the presence of the negatively charged phosphate skeletons in DNA^21^. The *R*_*ct*_ was further increased to ~ 10.5 kΩ during the hybridisation process. The increased *R*_*ct*_ value is due to the additional phosphate skeleton from the target DNA, inducing more electrostatic repulsion on the electrosurface. This proves the effective hybridisation of the target DNA on the immobilised device, forming a double-stranded DNA duplex on the device surface. The Nyquist plot was constructed for the immobilised electrode exposed to different concentrations of the target DNA (1 fM–1 nM) (Fig. 5b). The *R*_*ct*_ value increased with increasing concentrations of the target DNA upon hybridisation. This phenomenon is due to the increasing electrostatic repulsion between the negative ion and the negatively charged phosphate backbone found in the probe DNA, which led to the significant change in *R*_*c*t_^10^. To investigate the sensitivity of the AgNP-decorated LSG-NF biosensor, a linear correlation with the difference in the *R*_*ct*_ was plotted according to the equation:

$$ \Delta R_{ct} = Rct_{hybridization} {-}Rct_{immobilization} $$

corresponding to the logarithm of the complementary DNA concentrations (Fig. 5c). As shown in Fig. 5c, *ΔR*_*ct*_ increased linearly as the complementary DNA concentration increased. The differences in the values (*ΔR*_*ct*_) between the *R*_*ct*_ of the immobilized and hybridized species were found to fit the natural logarithm of the target DNA concentrations according to the linear relation: *ΔR*_*ct*_ = 0.812 × 10^3^x + 3.673 × 10^4^ with a regression coefficient (*R*^*2*^) of 0.9896.

### Bio-sensing analyses on Ag-decorated laser-scribed graphene nanofiber

We further investigated the analytical performance of the AgNP-decorated LSG-NF bioelectrode, as displayed in Fig. 5d–f. The electrode provided favourable linearity, with detection and quantification limits of ~ 10^−15^ M and ~ 10^−13^ M, respectively. The results were determined by estimating the signal-to-noise ratio as 3σ and 10σ, respectively^36^. The *R*_*ct*_ for the target DNA (1 nM) was ~ 39 kΩ, which is approximately 16 times greater than the *R*_*c*t_ of single-base mismatched DNA (~ 2.5 kΩ) at 1 nM, signifying that the biosensor has brilliant sequence sensitivity towards even a single-base mismatch. Thus, the obtained sensitivity is among the best outcomes reported^12^. Likewise, there was no complementation between the probe and triple mismatched DNA. The cross-reactivity with *Escherichia coli* and *Leptospira* species/serovars is shown in Fig. 5d. The *R*_*ct*_ values were not significantly affected by non-target substances, which suggests that non-target substances do not induce any specific interference. The repeatability of the analysis employing the AgNP-decorated LSG-NF bio-electrode was studied by comparing five samples from the same batch prepared under similar conditions. The repeatability curve for the developed bioelectrode is shown in Fig. 5e, which shows the relative standard deviation (RSD) obtained from five parallel measurements for samples prepared under the same processing conditions (3%). Finally, the stability of the developed bio-electrode was investigated by conducting a shelf-life study over a period of 8 weeks with storage at 4 °C; measurements were performed at a regular interval of 1 week. The results reveal that the developed biosensor is relatively stable and suffered from only 10% degradation of its activity throughout the 8 weeks of measurement (Fig. 5f).

## Conclusions

The presence of AgNPs in the LSG-NF bioelectrode created a novel biosensitivity-enhanced framework for the identification of *Mycobacterium tuberculosis*. The morphology of the unit was initially characterised by FESEM and TEM, which revealed that the prepared AgNPs were successfully accommodated on LSG-NF. Furthermore, LSG-NF was characterized by XRD and Raman spectroscopy prior to DNA immobilisation and hybridisation analysis. Added proof was provided by studying the surface chemical groups on the hybridised DNA surface. These findings clearly demonstrate the stability, repeatability, and selectivity up to the femtomolar level. Hence, this electrode could potentially contribute to the medical industry.

## Methods

### Materials and reagents

A 5 Mil polyimide (PI) film was provided by Dye Solar Cell Laboratory, Centre of Innovative Nanomaterials and Nanodevices (COINN), Universiti Teknologi PETRONAS (UTP). (3-Aminopropyl)trimethoxysilane (APTES) (≥ 98%; Sigma Aldrich, St. Louis, Missouri, USA), diluted in 2% ethanol as a solvent (CH_3_CH_2_OH; 95%; HmbG Chemicals, Germany) and silver nanoparticles were prepared using oil palm lignin and aqueous silver nitrate solutions (2.5% w/v AgNO_3_ in H_2_O, Sigma Aldrich; St. Louis, Missouri, USA) and were used for surface modification of the interdigitated electrode (IDE). Ethanolamine (C_2_H_7_NO; Sigma Aldrich; St. Louis, MO, USA) was used as the blocking agent. Phosphate-buffered saline (PBS) (1X; First BASE Biochemicals, Singapore) was used to maintain the optimal pH and improve the binding events during the impedance measurements. A 21-mer oligonucleotide with the following sequence was used in this study:

thiolated probe DNA (p-DNA): 5-CCG TGA TTT TCC TAA CTA AGG/3ThioMC3-D-3′; complementary target DNA (t-DNA):3′-GGC ACT AAA AGG ATT GAT TCC-5′; non-complementary target DNA (nc-DNA): 3′-G ATT CTG CCG CTT GGC TGC CAA-5′; one-base mismatched target DNA (m-DNA): 3′-GGC ACT AAA ATG ATT GAT TCC-5′; three-base mismatching target DNA (tm-DNA): 3′-GGC ATT AAA ATG ATT GGT TCC-5′ utilizing IS6110 gene sequences derived from the GenBank database. The single-strand thiolated probe DNA (p-DNA) was sought. The original genomic sequence was collected from GenBank under accession number AJ242908.1.

### Synthesis of laser-scribed graphene nanofiber (LSG-NF)

LSG-NF was synthesised on a polyimide (PI) film using a laser-scribing method. The PI film was cleaned using deionised (DI) water and attached to a glass piece for support. A CO_2_ laser (wavelength 680 nm) engraving system (V-460, Universal Laser System, Scottsdale, Arizona, USA) was used to convert the sp^3^ hybridized atoms of the PI film to sp^2^ hybridized carbon atoms. The adjustable parameters were the power, speed, and pulse per inch (PPI). The power limits the intensity of the laser that penetrates the PI film, whereas the speed controls the rate at which the nozzle travels in the X–Y direction, and the PPI determines the number of pulses with which the laser beam hits the PI film per inch. The final power, speed, and PPI used to synthesise LSG-NF were 100% (*P*_*max*_ = 30 W), 30%, and 500, respectively. The laser-scribing process was conducted in raster mode and performed under ambient conditions.

### Green synthesis of silver nanoparticles (AgNPs)

Aqueous silver nitrate solution (AgNO_3_, 250 mL, 1000 ppm) was combined with 250 mL (1000 ppm) of palm oil lignin and mechanically stirred for 2 h under atmospheric conditions. The mixture was centrifuged at 6000 rpm for 20 min. The residue was extracted and used for development of the biosensor (AgNPs).

### Fabrication of DNA bioelectrode

A 4 × 11 mm interdigitated electrode (IDE) containing six pairs of electrodes was drawn using CorelDraw software and fabricated by a laser-scribing process. The fabrication process was performed according to the parameters set in the previous step to prepare the laser-scribed graphene nanofibers.

### DNA immobilization and hybridization

The IDE was modified by AgNPs to ease covalent bond contact with the thiolated probe DNA. First, 5 μL of APTES was dropped on the IDE and incubated for 1 h, followed by 5 μL of AgNPs. Thereafter, 5 μL of thiolated probe DNA solution was dropped onto the IDE and incubated for 1 h, followed by rinsing with PBS. Target DNA with various concentrations (1 fM to 1 nM) was immobilised on the IDE for hybridisation analysis. In addition, non-complementary and mismatched DNA sequences were thoroughly studied.

### Experimental characterization

The surface morphology of the LSG-NF and AgNP-decorated LSG-NFs was evaluated by variable pressure (VP) FESEM (Carl Zeiss SUPRA55 VP, Gemini) analysis. A HITACHI HT 7830 high-transmission electron microscope (HRTEM) was used to obtain higher magnification images of LSG-NF and LSG-NF-AgNPs dispersed in ethanol and sonicated for 10 min before dropping on the copper grid. The structural properties and crystallisation of LSG-NF were studied using Raman spectroscopy (HORIBA Jobin Yvon HR800) and X-ray diffraction (X’Pert^3^ Powder and Empyrean, PANalytical with Cu-Kα radiation (λ = 1.54 Å)). XPS (Thermo Scientific K-Alpha) was used to analyse the immobilisation, hybridisation, and material composition, while FTIR (Perkin Elmer, Spectrum One) was used to analyse the functional groups present after surface modification and functionalization. Electrochemical impedance spectroscopy (EIS) measurements were performed using an Autolab PGSTAT302N by inserting the bioelectrode on an ECOPIA SPCB-01 sample mounting board. The EIS measurements were performed in the frequency range of 100 Hz–1 MHz with an AC amplitude of 0.01 V_RMS_, and the results were recorded at room temperature.

## Supplementary Information

Supplementary Information.

## Data Availability

Relevant data are included in the “Supporting Information”.
