# Supplementary material for: Laser-scribed graphene nanofiber decorated with oil palm lignin capped silver nanoparticles: a green biosensor
Source: Sci Rep. 2021 Mar 9;11:5475. doi: 10.1038/s41598-021-85039-2 (PMC7970908; doi:10.1038/s41598-021-85039-2)
Supplement: Supplementary file 1 — Supplementary Information. [file 41598_2021_85039_MOESM1_ESM.pdf]

Supplementary Information

Laser-Scribed Graphene Nanofiber Decorated with Oil Palm Lignin Capped Silver Nanoparticles: A Green Biosensor

Melvin Jia Yong Tai[1,2], Veeradasan Perumal[1,2,*], Subash C.B. Gopinath[3,4,*], Pandian Bothi Raja[5], Mohamad Nasir Mohamad Ibrahim[5], Iffah Najihah Jantan[5], Nur Syahirah Husnah Suhaimi[5], Wei-Wen Liu[3]

[1]Centre of Innovative Nanomaterials and Nanodevices (COINN), Universiti Teknologi PETRONAS (UTP), Seri Iskandar, Perak, Malaysia.

[2]Mechanical Engineering Department, Universiti Teknologi PETRONAS (UTP), Seri Iskandar, Perak, Malaysia.

[3]Institute of Nano Electronic Engineering, [4]Faculty of Chemical Engineering Technology, Universiti Malaysia Perlis (UniMAP), Perlis, Malaysia.

[5]School of Chemical Sciences, Universiti Sains Malaysia (USM), Gelugor, Penang, Malaysia.

* veeradasan.perumal@utp.edu.my and subash@unimap.edu.my

Formation of Silver Nanoparticles (AgNPs)

The formation of the AgNPs preliminarily confirmed by the gradual colour changes of the lignin solution. The colour of the lignin solutions changed from dark brown (Supplementary Figure 1a) to brownish black (Supplementary Figure 1b) immediately after addition of silver nitrate (AgNO3) solution. The change in colour indicate the reduction of the silver ion to AgNPs, which is aided by the lignin. The surface plasmon excitation of the AgNPs causes the colour changes of the solution [1]. This show that aqueous silver ions could be reduced by the lignin extract to produce a stable Ag NPs in water.

FESEM and EDX analysis

FESEM analysis is a form of surface imaging which entirely capable of determining the various particles sizes, distributions, shapes and the nanomaterial morphology. The interaction between electrons in the beam and the sample results in formation of signal used to obtain the information about the surface topography and composition [2]. The combination of FESEM and EDX analysis enable to determine the AgNPs powder morphology and the chemical composition. Supplementary Figure 2 shows the presence of AgNPs clearly formed from reduction process facilitated by lignin. The nanoparticles were mostly spherical in shape and below 100 nm in size. They are in agglomerated form due to the drying process from liquid before the analysis. Few individual particles were also observed. The elemental analysis of the AgNPs synthesized confirmed by EDX analysis as shown in Supplementary Figure 3. The spectrum shows a strong peak signal at 3 keV, a typical absorption of metallic AgNPs. The presence of other elements shows the impurities of the prepared nanoparticles. TEM analysis provides further insight on the morphology and size of the AgNPs synthesized.

TEM

TEM analysis is among the most important technique used for nanomaterial's characterization as it provides the quantitative measurements of particles, size distributions and its morphology. Supplementary Figure 4 shows the TEM images of AgNPs reduced by lignin. The silver nanoparticles predominated with spherical triangle, truncated triangle and ellipse. Majority of them are circular with smooth edges. The size of AgNPs obtained are around 18 nm to 53 nm.

UV-Vis Spectroscopy

The UV-Vis spectrum was observed to monitor the stability and the formation of the AgNPs. The unique optical properties of the AgNPs allowed them to interact with the specific wavelength of light [3]. The wavelength scale was fixed from 300 nm to 600 nm. The peak area formed between 400-500 nm proportional to with the increases of time from 0 min to 2 days. The absorbance increases as the time increase. This situation may cause by the increasing number of the AgNPs formation resulting from the reduction of silver ion present in the aqueous solution. There is a sharp peak around 450 nm (Supplementary Figure 5) indicate the formation of AgNPs. [4].

FTIR Spectroscopy

FTIR is the most suitable and simple technique used to determine and identify the active functional group in the lignin that involved in the formation of the AgNPs. As shown in Supplementary Figure 6, the lignin shows a wide band at 3500 to 3200 cm^{-1} assigned to the OH stretching due to the presence of the alcoholic and phenolic hydroxyl group in the lignin structure. The absorption band occurs at 1760 to 1665 cm^{-1} due to the stretching vibration of carbonyl compounds. The intense peak at 1190, 1088, 1048 cm^{-1} originates from methoxy groups. The absorption bands in the region ranging from 900 to 700 cm^{-1} may due to the deformation vibrations of C-H bond on the benzene ring. These functional group show a decreasing in intensities after the reduction reaction to form AgNPs. This indicate the reduction process aided by these functional group.

Electrochemical Impedance Spectra of AgNPs

The EIS of AgNPs were determined by dropping 10 μL of AgNPs onto a screen-printed circuit electrode (SPCE). Supplementary Figure 9 revealed the measurement of bare SPCE and AgNPs, the Rct value of bare SPCE was obtained at ~ 12 kΩ. The Rct were decreased to ~ 650 Ω when AgNPs were added. The inset shows the magnified spectra of the EIS obtained along with the repeatability curve of 3 samples prepared under similar conditions.

Supplementary Figure

Supplementary Figure 1

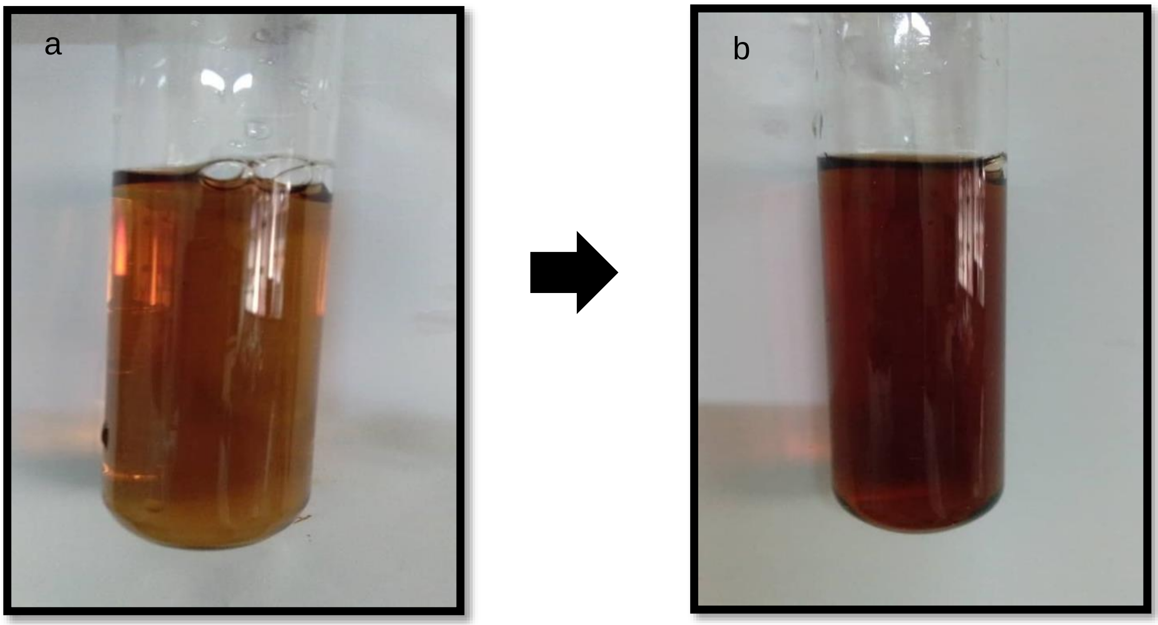

Supplementary Figure 1: Colour changes of lignin (a) before and (b) after adding $AgNO_3$ for 2 days.

Supplementary Figure 2

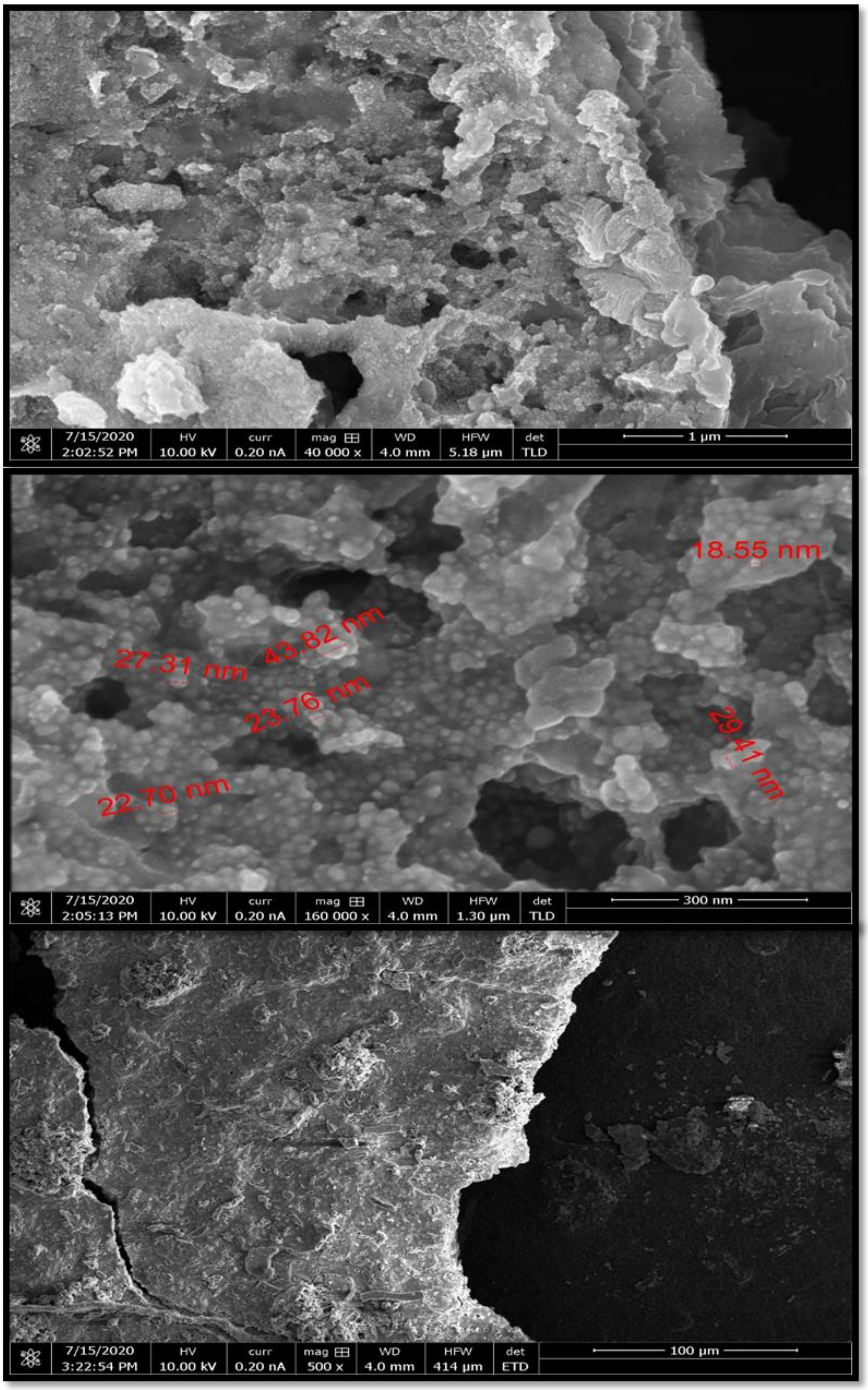

Supplementary Figure 2: FESEM images of lignin reduced AgNPs with magnifications of (a) 40Kx, (b)160Kx and (c) 500x.

Supplementary Figure 3

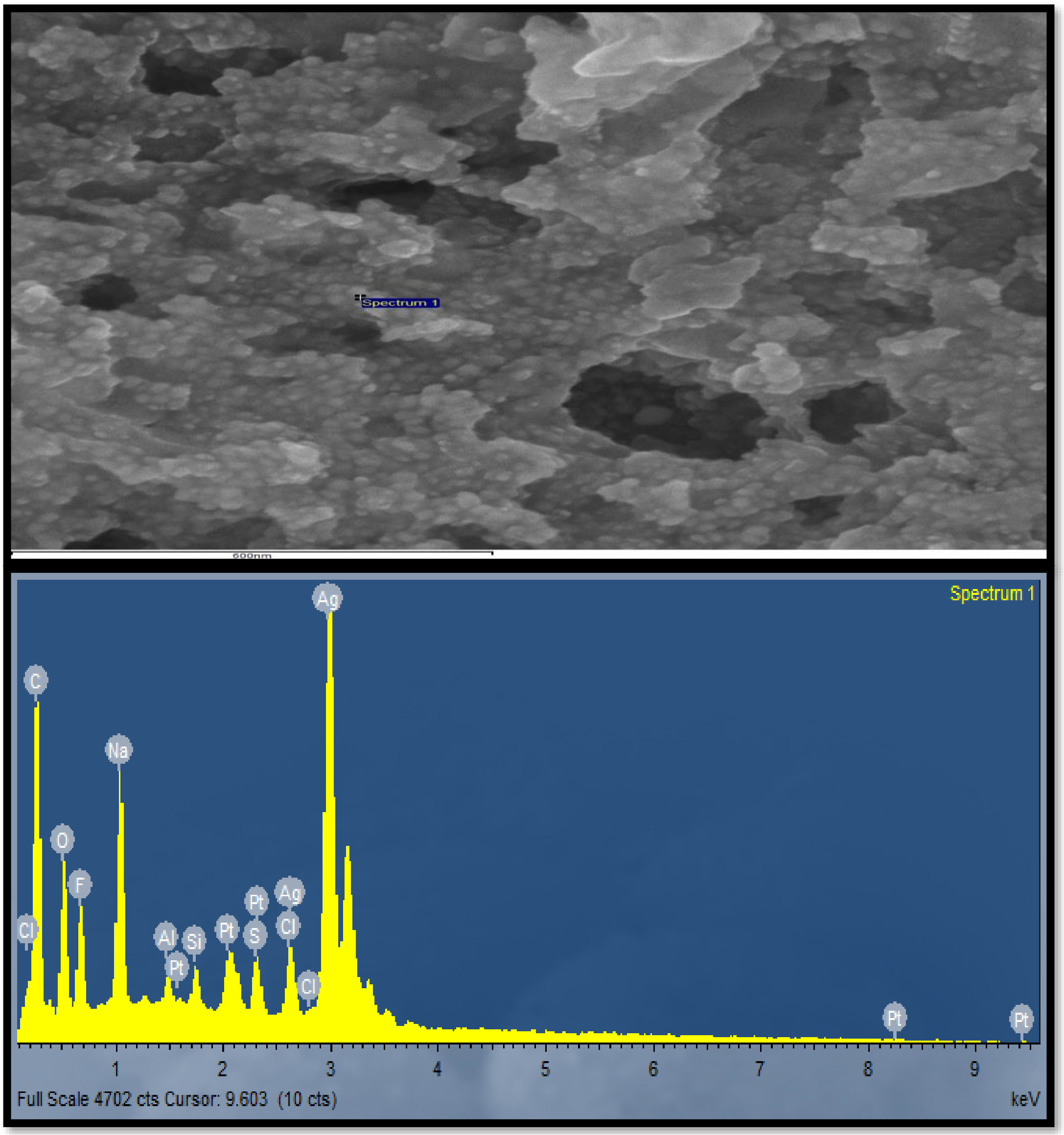

Supplementary Figure 3: EDX spectrum of lignin reduced AgNPs.

Supplementary Figure 4

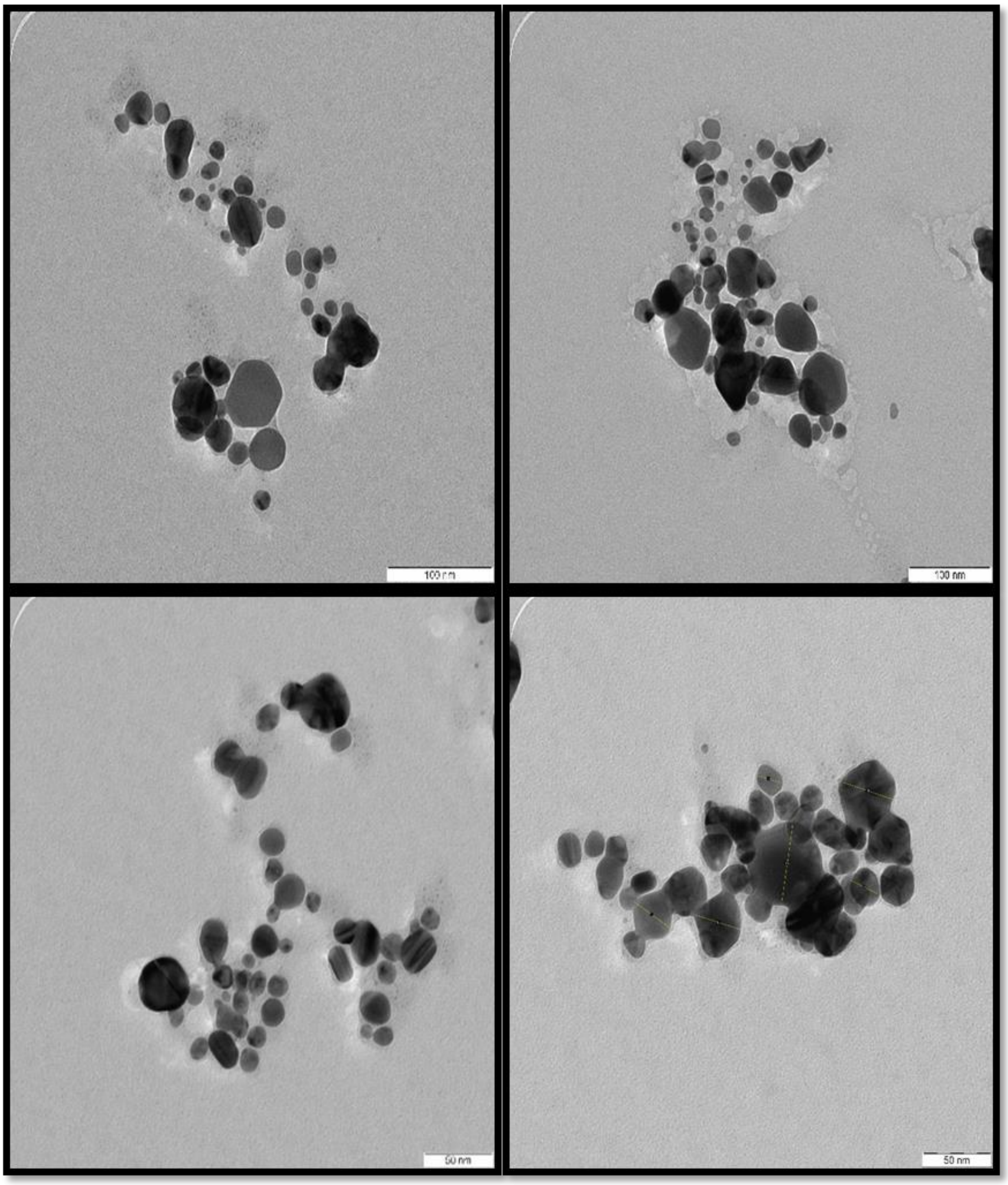

Supplementary Figure 4: TEM images of lignin reduced AgNPs.

Supplementary Figure 5

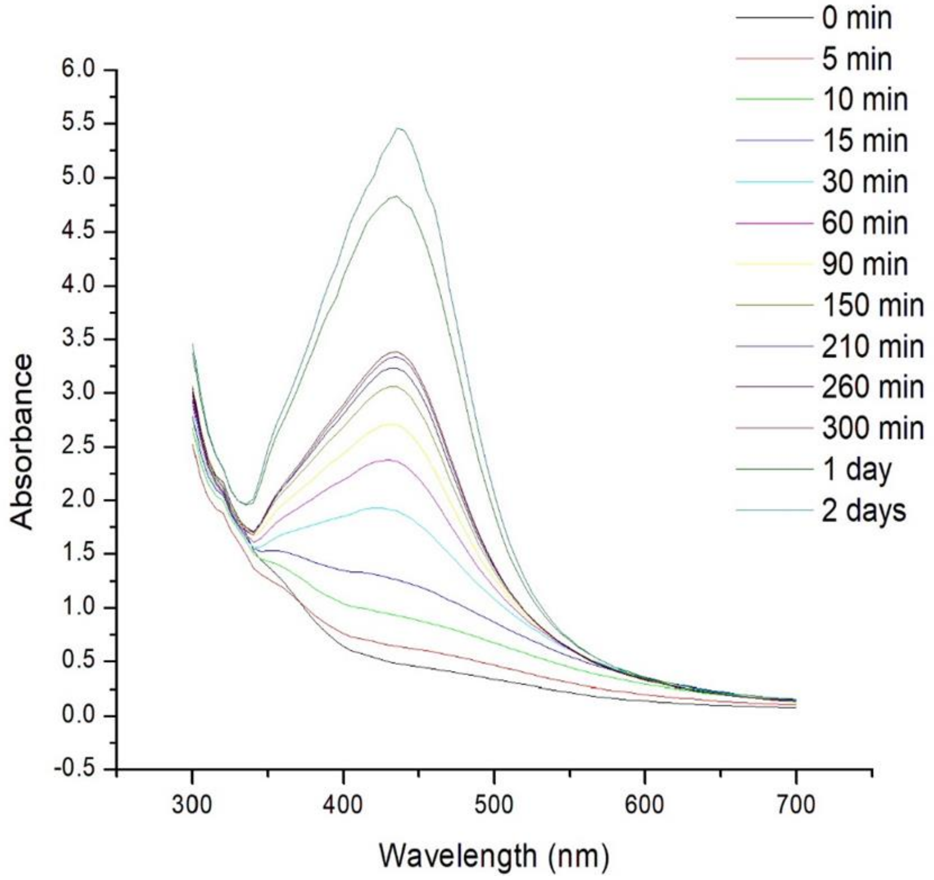

Supplementary Figure 5: UV-Vis Spectrum of lignin extract reduced AgNPs at different time intervals.

Supplementary Figure 6

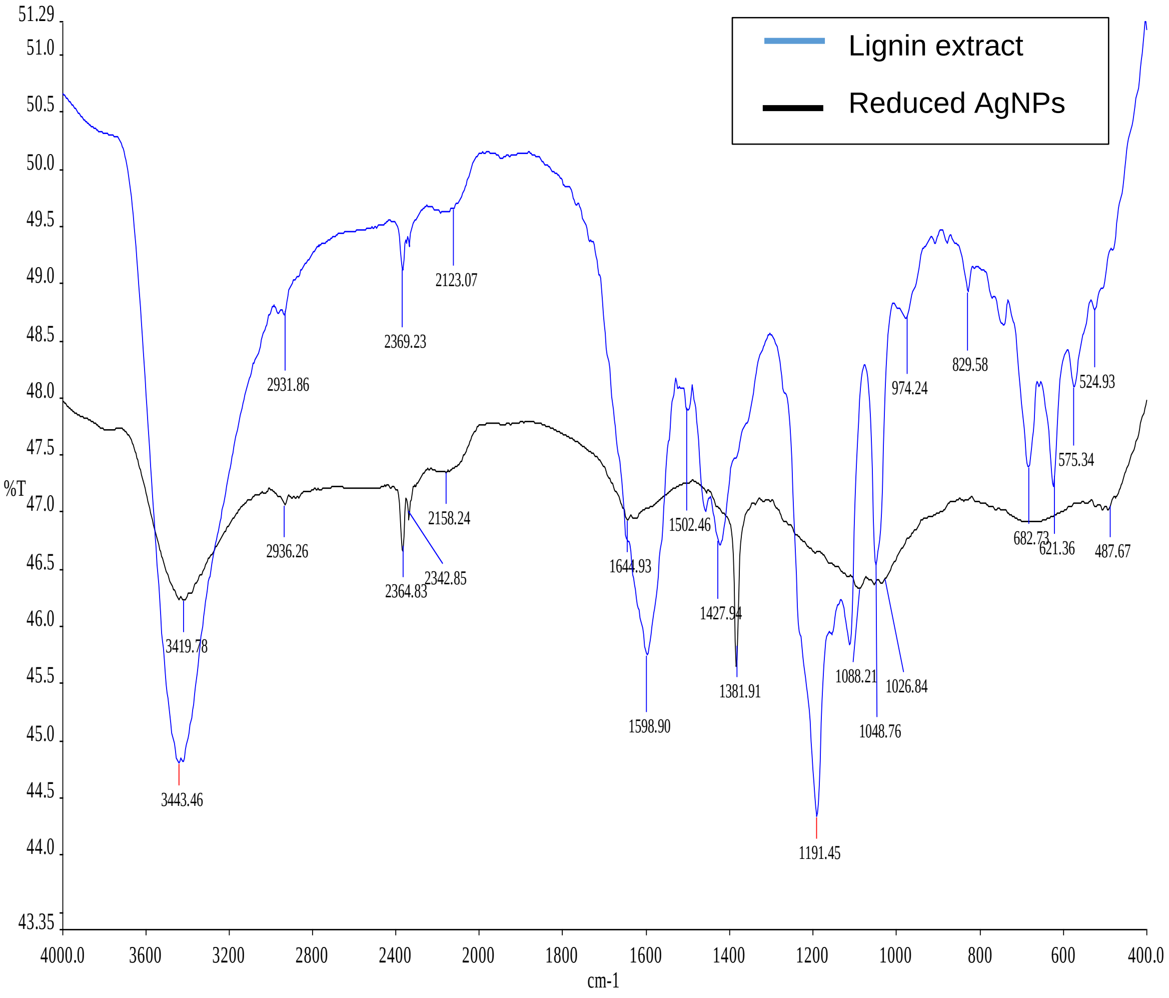

Supplementary Figure 6: FTIR spectrum of lignin (blue) and reduced AgNPs (black).

Supplementary Figure 7:

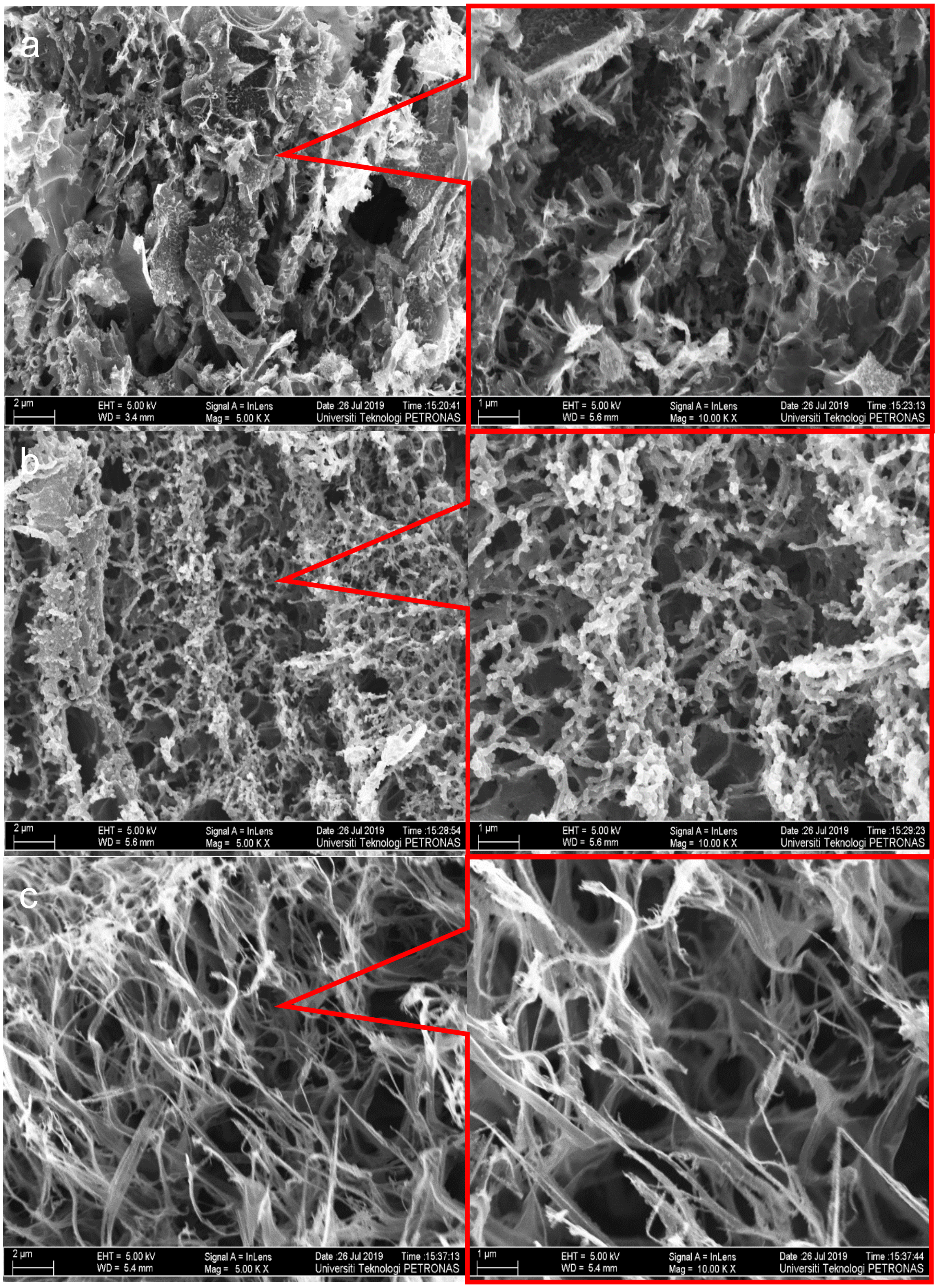

Supplementary Figure 7: FESEM images of laser scribed graphene synthesized at (a) 50%, (b) 70 % and (c) 100% showing the formation of nanofibers upon increasing the 30W CO_2 laser power.

Supplementary Figure 8:

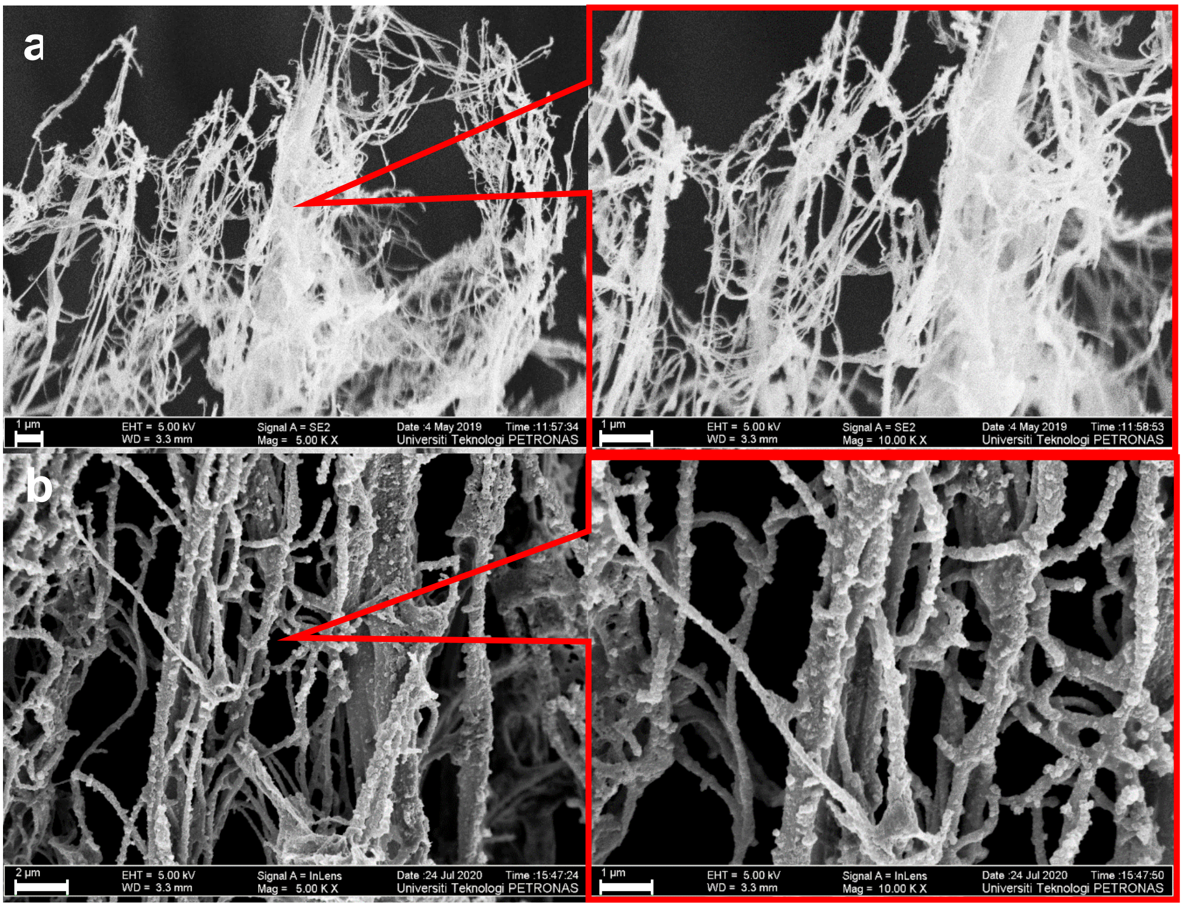

Supplementary Figure 8: FESEM image of (a) Laser Scribed Graphene Nanofibers revealing the fine hair-structure of graphene nanofibers obtained with 30W CO_2 laser. (b) FESEM image of Laser Scribed Graphene Nanofibers after accommodated by AgNPs resulting coral-structure obtained.

Supplementary Figure 9:

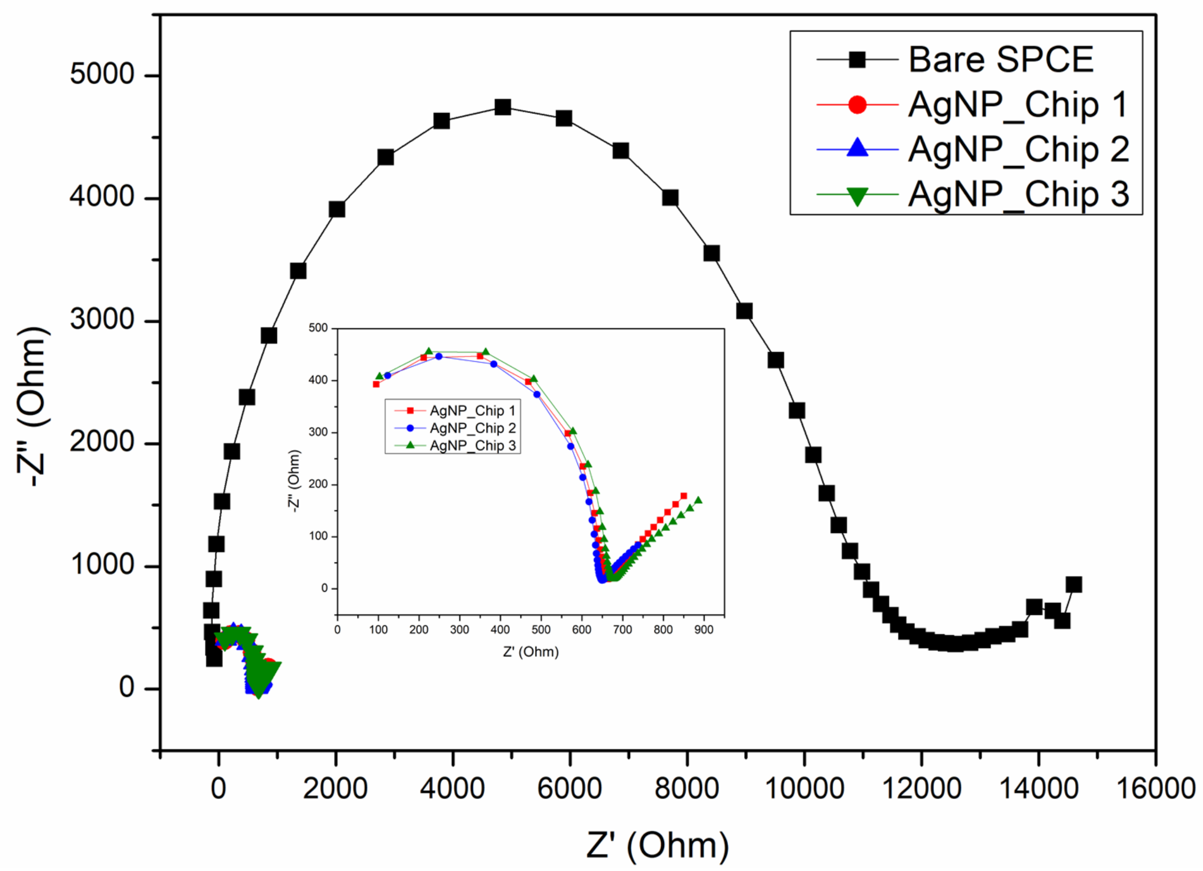

Supplementary Figure 9: EIS measurement of bare SPCE (black) and AgNPs. The inset shows the magnified spectra and the reproducibility curve of the AgNPs modified SPCE.
